# HIIE Protocols Promote Better Acute Effects on Blood Glucose and Pressure Control in People with Type 2 Diabetes than Continuous Exercise

**DOI:** 10.3390/ijerph19052601

**Published:** 2022-02-24

**Authors:** Gabriela de Oliveira Teles, Paulo Gentil, Lucas Raphael Bento e Silva, Wátila de Moura Sousa, Camila Simões Seguro, Ana Cristina Silva Rebelo

**Affiliations:** 1 College of Physical Education and Dance, Federal University of Goias, Campus Samambaia, Goiânia 74690-900, Brazil; 2 Department of Physical Education, Faculdade Araguaia, Goiânia 74223-060, Brazil; lucasraphaelbs@gmail.com; 3 Faculty of Medicine, Federal University of Goias, Goiânia 74605-050, Brazil; watilams@gmail.com; 4 Faculty of Nutrition, Federal University of Goias, Goiânia 74605-080, Brazil; miaseguro@gmail.com; 5 Department of Morphology, Institute of Biological Sciences, Federal University of Goias, Goiânia 74690-900, Brazil; anacristina.silvarebelo@gmail.com

**Keywords:** hyperglycemia, interval training, blood pressure, physical exercise, heart rate

## Abstract

This study compared the acute effects of a session of different high-intensity interval exercise (HIIE) protocols and a session of moderate-intensity continuous exercise (MICE) on blood glucose, blood pressure (BP), and heart rate (HR) in people with Type 2 Diabetes Mellitus (DM2). The trial included 44 participants (age: 55.91 ± 1.25 years; BMI: 28.95 ± 0.67 kg/m^2^; Hb1Ac: 9.1 ± 2.3%; 76 mmol/mol) randomized into three exercise protocols based on the velocity at which maximum oxygen consumption was obtained (vVO2 max): long HIIE (2 min at 100% vV̇o2peak + 2 min of passive rest); short HIIE (30 s at 100% vV̇o2peak + 30 s of passive rest); or MICE (14 min at 70% vV̇o2peak) on a treadmill. Capillary blood glucose, BP, and HR measurements were taken at rest, during peak exercise, immediately after the end of exercise, and 10 min after exercise. Long and short HIIE protocols reduced capillary blood glucose by 32.14 mg/dL and 31.40 mg/dL, respectively, and reduced systolic BP by 12.43 mmHg and 8.73 mmHg, respectively. No significant changes were observed for MICE. HIIE was found to promote more acute effects than MICE on glycemia and BP in people with DM2.

## 1. Introduction

Diabetes mellitus type 2 (DM2) is a chronic metabolic condition characterized by high blood glucose levels due to impaired insulin sensitivity and associated with autonomic dysfunction, retinopathy, neuropathy, nephropathy, and cardiovascular diseases, among other complications [1]. In this regard, cardiovascular diseases are the most common cause of death among people with diabetes mellitus [2]. Therefore, controlling risk factors such as blood glucose and blood pressure (BP) is essential for reducing cardiovascular complications during both rest and effort [3]. 

Non-pharmacological treatments that involve lifestyle changes, such as regular physical exercise, are effective strategies for controlling and preventing DM2, leading to reductions in glycated hemoglobin (HbA1c) levels, blood glycemia [4,5], and BP [6]; increases in insulin sensitivity [7] and cardiorespiratory fitness [8]; and improved lipid profile [9]. However, as DM2 is usually accompanied by other performance-limiting clinical conditions, there is a need for a comprehensive discussion regarding the type, intensity, and duration of exercise for this population, taking cost–benefit analysis into consideration [10].

Although most guidelines recommend moderate-intensity continuous training, some studies have shown that high-intensity interval exercise (HIIE) has positive effects on the cariometabolic risk factors of people with DM2 [11,12]. This type of training can induce similar cardiometabolic adaptations and, in some cases, proves even better than moderate-intensity continuous exercise (MICE), especially in improving glycemic control, glycated hemoglobin, and the cardiorespiratory fitness of people with DM2 [7,10,13,14]. 

Previous studies have shown that different HIIE protocols have different impacts on acute and chronic responses, which makes it necessary to analyze HIIE considering its specific characteristics, instead of drawing general conclusions [15]. Among the variables that can be manipulated during HIIE, the duration of exercise has been shown to have an important impact on cardiovascular stress [16,17]. Even when the intensity and amount of exercise are kept constant, reducing the duration of this exercise seems to reduce the cardiovascular risk, suggesting that short HIIE (with a duration of 1 min or less) can promote a lower heart rate than MICE, even when it is performed at higher intensities [16,18,19]. However, these studies are limited to young healthy people.

Thus, given the controversies and the scarcity of studies investigating the acute effects of different HIIE and MICE protocols in people with DM2, the objective of this study was to investigate and compare the acute effects of one session of different HIIE protocols and one session of MICE on the capillary blood glucose, blood pressure, and heart rate of people with DM2.

## 2. Materials and Methods

### 2.1. Participants and Procedures

Patients were recruited from the 3rd Diabetes Marathon promoted by the Eye Bank Foundation of the State of Goiás, Brazil, in November 2018. The inclusion criteria were patients having been diagnosed with DM2, over 40 years old, and not having participated in any physical training program for at least 6 months. Patients with self-reported infectious disease; self-reported smoking; arrhythmias, angina, and frequent extrasystoles; severe lung diseases; and self-reported musculoskeletal and cardiovascular problems that could impair the evaluation were excluded from the study.

### 2.2. Data Collection

Data collection took place in three visits. The first involved an interview and blood collection; the second involved anthropometric and hemodynamic evaluations and the cardiopulmonary exercise test; and the third involved physical exercise sessions.

During the first visit, the volunteers completed a questionnaire to capture their personal data, clinical history, disease progression, and the medications they used. Blood collection was then performed after 12 h of fasting. Their fasting blood glucose and HbA1c dosage were evaluated to confirm the diagnosis. Their fasting blood glucose was evaluated according to the enzymatic method using LABTEST kits and the LABMAX PLENNO equipment [20]. A glycated hemoglobin kit was used to measure their HbA1 dosage, using the colorimetric test (Laborclin, Pinhas, Paraná). On a different day, the patients had their cardiac and pulmonary auscultation and resting BP and HR measured using an automated oscillometric sphygmomanometer (Omron HEM-705) following previous recommendations [21]. The patient rested seated for 10 min before each measurement was taken. During measurement, the patient’s shoulder was flexed and their elbow was extended to the level of their heart. During the anthropometric assessments, patients remained barefoot and wore light clothing. Their body mass index (BMI) was calculated by dividing their body mass by their height measured in meters squared (kg/m^2^) [22]. During the first visit, patients were instructed to avoid radical changes in their diet until the day of the exercise session in order to prevent bias in glycemic control. 

A cardiopulmonary exercise test was used to identify possible changes in hemodynamic, ventilatory, and cardiovascular responses to physical exertion using a ramp-type load increment protocol with a treadmill (Micromed^®^, Centurion 200, Brasília, Brazil) and gas analyzer (Cortex analyser^®^ Metalyser II, Rome, Italy). The test started with a two-minute warm-up and then the speed was increased by 0.1 km/h every 10, 20, or 30 s until exhaustion, without inclination. The test was followed by a four-minute recovery period. The patients’ heart rate was continuously monitored using a heart monitor (Polar v800, Kempele, Finland) and their blood pressure was measured by Korotkoff auscultation with a mercury sphygmomanometer (WanMed, São Paulo, SP, Brazil) and a stethoscope (Littman, São Paulo, MN, USA). The test was supervised by a trained professional and it was interrupted if the patient experienced strige discontinuity or reached their predicted maximum heart rate or a respiratory exchange ratio >1.15 [23].

The velocity at which the volunteers reached peak oxygen consumption (vV̇o2peak) was used to determine the amount of exercise they were prescribed. 

### 2.3. Exercise Sessions

The exercise sessions were conducted in a public hospital. The patients were randomized among three protocols adapted from previous studies [16,24,25]. The patients who were assigned the long HIIE protocol carried out five repetitions of 2 min at 100% of vV̇o2peak, with 2 min of passive recovery; patients assigned the short HIIE carried out 20 reps of 30 s at 100% vVo2max, with 30 s of passive recovery; and those assigned MICE carried out 14 continuous minutes at 70% of vV̇o2peak. All the protocols included a warm-up and a cool-down of 2 min at 50% of vV̇o2peak. Familiarization sessions were carried out twice a week during two consecutive weeks, with characteristics similar to those of the data collection.

**Figure 1:**
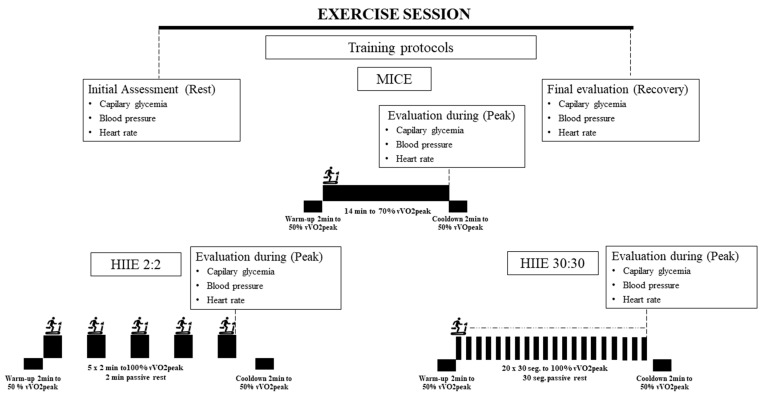
Diagram of training protocols in the session. HIIE: high-intensity interval exercise; MICE: moderate-intensity continuous exercise; vVo2max: velocity relative to the maximum volume of oxygen.

The testing sessions took place during the third week. Before the evaluation, the patients remained seated for 10 min and had their blood glucose, BP, and HR measured. Then, each patient performed a physical exercise session, and the same measurements were repeated 10 min after the test. Their capillary blood glucose was measured using the AccuCheck Perfoma glucometer, using the index finger. BP and HR were measured using the Omron 7122 automatic sphygmomanometer. Their central (“cardiorespiratory”) and peripheral (“muscular”) RPE were monitored using the adapted Borg Scale (0 to 10). We opted to separate the RPE because our group had shown that people with high levels of blood glucose might demonstrate an unmatched response between muscular and cardiac responses [26,27]. Figure 1 shows the characteristics of the training protocols and session logistics.

### 2.4. Data Analysis

Two-way ANOVA with repeated measurements was performed for intra-group and between-group comparisons. Repeated measures were used, with the confidence interval adjusted by the Bonferroni method for post hoc comparisons. The effect size was calculated by η^2^. The level of significance was *p* ≤ 0.05. Data were analyzed in the Statistical Package for Social Sciences (SPSS—IBM Corp, Armonk, NY, USA), version 2.0.

## 3. Results

A total of 44 individuals with a mean time of diagnosis of 11.98 ± 6.46 years participated in this study. The medications most commonly used by the participants were biguanide (Metformin—40%; Glifage—17.8%), diuretics (Hydrochlorothiazide—28.9%), angiotensin receptor antagonists (Lozartana—22.2%; Aradois—15.9%), and statins (Simvastatin—6.7%). Most participants were overweight according to BMI classification >25.0 kg/m^2^ (*n* = 35, 79.5%). The other sample characteristics are presented as means ± standard deviations in Table 1. One-way ANOVA showed no significant differences between groups for any variable before evaluation (*p* > 0.05) 

The mean treadmill speed and RPE for each group are presented as mean ± standard deviation in Table 2. RPE was significantly higher for long HIIE than short HIIE and MICE.

Comparisons between patients’ cardiovascular variables and blood glucose at rest, peak, and recovery are presented as means ± standard deviations along with pre-post variations (Δ) and effect sizes (η^2^) in Table 3.

There was an increase in SBP at the peak of the training session in all groups. However, the values reduced beyond the basal state in both HIIE groups, with greater decreases seen for long HIIE (*p* < 0.05). DBP did not show significant changes for any protocol. HR significantly increased at the peak of the exercise in all groups and was higher for both HIIE groups than for MICE.

Blood glucose significantly reduced only at peak exercise for MICE. There was a reduction in blood glucose from post- to pre-test for the HIIE groups, with a greater reduction seen for long HIIE (Δ = 32.14 mg/dL). Effect sizes were large (η^2^ > 0.14) for the HR values in the long HIIE groups, intermediate (0.06 < η^2^ < 0.11) for the SBP values in the HIIE groups, and for HR in the short HIIE and MICE groups.

## 4. Discussion

The present study aimed to investigate the acute effects of different HIIE and MICE protocols on capillary blood glucose, BP, and HR in people with DM2. Long and short HIIE sessions reduced capillary blood glucose by 32.14 mg/dL and 31.40 mg/dL after exercise, while glycemia significantly decreased during MICE (21.14 mg/dL) and tended to increase during HIIE. This information might be important for glucose monitoring and diet adjustment. For example, if glycemia is low before HIIE, it might be interesting to evaluate the need for glucose ingestion after exercise or to adjust medication dose or timing in exercise days to avoid hypoglycemia. The lowest glucose levels during MICE also might have applications for medication and diet adjustments, since it might be necessary to ingest glucose or to reduce medication dosage before exercise. This might also help to determine the type of exercise best suited to the patient’s current state. To avoid hypoglycemia during exercise, HIIE should be chosen; however, to avoid hypoglycemia after exercise, MICE should be chosen. 

These results might also have an impact on clinical aspects, since regular exercise sessions might help in glycemic control, which is a critical objective in DM2 treatment as it reduces the incidence of related complications, including the risk of cardiovascular events [13]. In this regard, our study corroborates previous studies which showed reductions of 40 mg/dL immediately after exercise, lasting for up to 6 h after and reaching reductions of 60 mg/dL [28]. In a more prolonged analysis, Gillen et al. showed that a single session of HIIE reduced the mean 24 h glucose and postprandial glucose in people with DM2 [11]. 

The differences found in glycemic response between exercise modes are in agreement with previous studies and might be related to the physiological impact of different exercise intensities and their interactions with the medications used [29,30]. Lower-intensity activity has a higher dependence on the glucagon/insulin axis for controlling blood glucose, which might be affected by medications such as insulin and biguanides. However, higher-intensity physical activities had a higher impact on the sympathetic system and depended more on catecholamines, which are not affected by the most common hypoglycemic medications. 

In patients with DM2, HIIE is usually associated with a transient increase in blood glucose levels, which occurs because, during exercise, there is a greater degradation of hepatic glycogen (glycogenolysis). This degradation makes glucose available to the bloodstream, resulting in an acute increase in capillary blood glucose [30]. However, there was no such increase in blood glucose at peak exercise in the present study, only a progressive reduction, as in the studies by Mendes et al. [14] and Santiago et al. [6]. The hypothesis for this finding is that the initial glycemic values were already very high, which might have prevented further increases.

After physical exercise, there was a reduction in blood glucose, which might be associated with increased blood flow to the patients’ muscle fibers and improvement in their mitochondrial function, increasing tissue sensitivity to insulin and, therefore, glucose uptake in muscles and adipocytes [3]. In addition, there was an increase in the activity of glycolytic and oxidative enzymes [6]. 

As for cardiovascular stress markers, SBP increased similarly during exercise, but reduced by 12.43 mmHg and 8.73 mmHg during recovery from long and short HIIE, respectively. Although the acute increases might reflect an increased risk, the exercise hypotensive response might have important clinical applications, since it is associated with long-term benefits in BP reduction [31,32,33,34]. In this sense, the reduction in SBP has important clinical implications for treating people with DM2 because controlling BP contributes to alleviating microvascular and macrovascular risks. There was also an increase in HR at peak exercise in both HIIE protocols, while the MICE group did not show significant HR changes. Therefore, a single session of HIIE, either long or short, might provide more acute cardiovascular stress than MICE, but have a more pronounced effect on post-exercise hypotension. This information is important for a cost–benefit analysis. If the patient’s cardiovascular risk is high, it is recommended to be more conservative and propose MICE; however, it the risk is controlled, then HIIE might be chosen for its potentially higher benefits.

Long HIIE had significantly higher RPE values when compared to short HIIE and MICT. Central RPE is related to respiratory-metabolic effort, and closely related to ventilation, oxygen consumption, and HR, among other physiological mediators. Peripheral SBP, on the other hand, refers to the local effort related to metabolic acidosis, regional blood perfusion, and energy substrates [13,18]. Therefore, the present results showed that long HIIE is the most strenuous, requires the most effort, and results in a high recovery-rest variation, which must also be considered during exercise prescription to avoid attrition, since exercise adherence is associated with the reductions in glycated hemoglobin [35]. 

This study of the acute responses to different exercise models support the results of different randomized clinical trials that analyzed skeletal muscles [27], the vascular system, respiratory changes [24], cardiac function [36,37], exercise capacity [38], inflammation, quality of life [36], and other physiological markers such as V̇O2peak and endothelial function, with greater improvements seen for the HIIE protocols compared to MICE [8,24,36]. However, it is important to test to chronic adaptation to different protocols in order to see if these acute effects are reflected in long-term changes.

## 5. Conclusions

Our results provide important information for exercise prescription, taking cost–benefit analysis into consideration. Based on the acute responses, it can be concluded that HIIE, especially long HIIE, might promote the best clinical outcomes; however, it is also associated with higher perceived effort, which can increase the risk of attrition and acute events. On the other hand, although MICE was associated with lower beneficial responses, it was also the exercise type with lower risk factors and lower effort perception. Therefore, MICE could be used during the adaptation phase and for patients at higher risk. On the other hand, HIIE could be used for progression and when the risk factor is controlled to obtain better clinical results. Moreover, during the analysis of acute effects, it might be of clinical importance to adjust a patient’s diet and medication. In this regard, it would be important to monitor their blood glucose after HIIT to determine the need to increase glucose ingestion or decrease medication dose when performing protocols that decrease blood sugar.

### Limitations

Due to the nature of the study, it was not possible to use a blind methodology. Other limitations were the sample size and the absence of a longer follow-up after the exercise sessions. The present study involved a between-subject comparison; therefore, it cannot account for interindividual differences in exercise responses. For that, it would be necessary to perform each type of exercise and use a within-subject design to evaluate the potential effects in a more rigorous manner. However, we opted for this design in order to avoid the effects of repeated exercise bouts. 

## Figures and Tables

**Table 1:** Characteristics of patients with DM2 classified by group.

	HIIE Long (*n* = 14)	HIIE Short (*n* = 15)	MICE (*n* = 15)	TOTAL (*n* = 44)
Age (years)	54.64 ± 8.91	55.67 ± 7.44	57.33 ± 8.93	55.91 ± 1.25
Weight (kg)	80.65 ± 14.52	79.45 ± 10.95	76.27 ± 16.91	78.75 ± 21.30
BMI (kg/m^2^)	29.44 ± 4.94	28.94 ± 3.64	28.49 ± 4.94	28.95 ± 0.67
Blood glucose (mg/dL)	142.43 ± 59.06	126.47 ± 38.23	133.80 ± 54.84	134.05 ± 7.62
Hb1ac (% mmol/mol)	9.6 ± 2.9; 81	8.9 ± 1.6; 74	9.0 ± 2.4; 75	9.1 ± 2.3; 76
HR (bpm)	75.0 ± 7.38	66.0 ± 9.35	70.75 ± 9.39	71.11 ± 9.07
SBP (mmHg)	143.57 ± 23.65	131.07 ± 14.24	131.0 ± 15.12	135.12 ± 18.62
DBP (mmHg)	90.14 ± 12.24	83.47 ± 9.19	87.50 ± 8.69	86.95 ± 10.28

HIIE: high-intensity interval exercise; MICE: moderate-intensity continuous exercise; BMI: body mass index; Hb1ac: glycated hemoglobin; HR: heart rate; SBP: systolic blood pressure; DBP: diastolic blood pressure.

**Table 2:** Mean values of speed and subjective perception of exertion of patients with DM2 organized by group.

	HIIE Long (*n* = 14)	HIIE Short (*n* = 15)	MICE (*n* = 15)	TOTAL (*n* = 44)
Velocity (km/h)	8.22 ± 0.56	7.32 ± 0.38	5.19 ± 0.43	6.88 ± 2.14
Central SPE	7.5 ± 1.02 *	5.47 ± 2.0	5.33 ± 2.29	6.07 ± 2.07
Peripheral SPE	7.79 ± 1.37 *	5.80 ± 2.37	5.80 ± 1.74	6.43 ± 2.06

* *p* < 0.05, values with significant differences when compared to the other groups.

**Table 3:** Comparison of cardiovascular variables and blood glucose at rest, peak, and recovery from the evaluation of patients with DM2 organized by group.

		Resting	Peak	Recovery	∆	η^2^
HIIE long		134.21 ± 19.95	159.93 ± 6.72	121.79 ± 14.68	−12.43 *	0.11
HIIE short	SBP	123.60 ± 12.72	154.0 ± 4.96	114.87 ± 9.08	−8.73 *	0.13
MICE		125.33 ± 15.56	155.0 ± 7.22	124.87 ± 17.30	−0.47	0.00
HIIE long		134.21 ± 19.95	159.93 ± 6.72	121.79 ± 14.68	−12.43 *	0.11
HIIE short	DBP	123.60 ± 12.72	154.0 ± 4.96	114.87 ± 9.08	−8.73 *	0.13
MICE		125.33 ± 15.56	155.0 ± 7.22	124.87 ± 17.30	−0.47	0.00
HIIE long		77.57 ± 9.33	133.93 ± 11.70	89.93 ± 12.04	12.35 *	0.24
HIIE short	HR	79.60 ± 10.70	124.53 ± 6.55	92.07 ± 23.41	12.47 *	0.10
MICE		76.6 ± 12.25	105.0 ± 9.30	84.73 ± 13.32	8.13	0.09
HIIE long		172.86 ± 77.33	161.29 ± 77.05	140.71 ± 72.61	−32.14 *	0.04
HIIE short	Glucose	168.67 ± 73.88	152.33 ± 68.12	137.27 ± 69.31	−31.40 *	0.04
MICE		148.13 ± 43.99	126.80 ± 44.0	143.07 ± 56.80	−5.07	0.00

HIIE: high-intensity interval exercise MICE: moderate-intensity continuous exercise; SBP: systolic blood pressure; DBP: diastolic blood pressure; HR: heart rate; ∆: rest–recovery variation; η^2^: effect size. Values are expressed as means and standard deviations. * *p* < 0.05, values with significant differences.

## Data Availability

Data are available on Google Drive. The data presented in this study are openly available at: https://drive.google.com/drive/folders/1PdUEMd5UlJUwjU1Gi-rq5rVI7Woiy461?usp=sharing (accessed on 12 January 2022) under the title artigo HIIT.
